# *Escherichia coli* K-12 Transcriptomics for Assessing the Mechanism of Action of High-Power Ultrasound

**DOI:** 10.3390/microorganisms11112768

**Published:** 2023-11-14

**Authors:** David Spiteri, Sholeem Griffin, Kimon Andreas Karatzas, Christian Scerri, Vasilis P. Valdramidis

**Affiliations:** 1 Department of Food Science and Nutrition, University of Malta, MSD 2080 Msida, Malta; dspit010@gmail.com (D.S.); sholeem.griffin@um.edu.mt (S.G.); 2 Centre for Molecular Medicine and Biobanking, University of Malta, MSD 2080 Msida, Malta; christian.scerri@um.edu.mt; 3 Department of Food and Nutritional Science, University of Reading, Reading RG6 6EU, UK; k.karatzas@reading.ac.uk; 4 Department of Physiology and Biochemistry, University of Malta, MSD 2080 Msida, Malta; 5 Department of Chemistry, National and Kapodistrian University of Athens, 34400 Psachna, Greece

**Keywords:** ultrasound, sonochemistry, *E. coli* K-12 mutants, *GadW*, RNA-Seq, transcriptomics

## Abstract

An investigation into the mechanisms of action on bacteria involving exposure to stress factors was conducted in this study. The effects of ultrasound on *Escherichia coli* K-12 MG1655 and its isogenic mutant, ∆*gadW*, under high power ultrasound treatments (26 kHz) were screened and identified by analysing their transcriptome differences between primary and secondary sequential treatments using RNA-Seq. This also helped to assess any developed protection for cells between different generations. According to our results, 1825 genes of all tested conditions were expressed, playing different roles in the cell. The expression of these genes is associated with DNA damage, cell membrane integrity, and also metabolic effects. The studied strains also showed different differential expressed genes (DEGs), with some genes being directly responsible for defence mechanisms, while others play an indirect effect due to cell damage. A gradual decrease in the expression of the genes, as we moved from just one cycle of ultrasound treatment to sequential treatment, was evident from a heat map analysis of the results. Overall, *E. coli* K-12 builds a self-protection mechanism by increasing the expression of genes involved in the respiration for increased growth, and production of flagellum and pili. It can be concluded that high power ultrasound is a technology that triggers several different defence mechanisms which directly link to *E. coli*.

## 1. Introduction

The application of ultrasonic technology has most recently received wide attention in water and wastewater treatment and environmental remediation areas in relation to disinfection procedures [1,2]. Such applications include sonochemistry, where ultrasound is used for the acceleration of chemical reactions or extraction of specific chemical compounds, dispersion and disruption of biological cells, removal of trapped gasses and others [3,4,5,6,7]. This is especially important in wastewater treatment plants, where a combination of ultrasound effects, like the disruption of bacterial and colloidal particles, or disintegration of algal blooms, can assist in the efficiency of treatments [3,5,6].

Ultrasound generates elastic vibrations and waves whose frequency is over 15–20 kHz. While ultrasound can stimulate the activity and growth of microorganisms at low intensities and short influence durations, at greater intensities it kills and/or inactivates microorganisms. Long term water treatment by ultrasound of 20–100 kHz with a sound intensity of between 10 and 1000 W/cm^2^ can result in effective disinfection applications [8]. The disinfection capacity of sonication in water is due to the phenomenon of acoustic cavitation, which is the formation and collapse of microbubbles occurring in milliseconds that produce extreme temperature and pressure gradients [4,9]. Indeed, the collapse of these microbubbles leads to extremely high local temperatures and pressures. These conditions have been shown to result in the generation of highly reactive radicals, such as OH· and H·. Ultrasound is, therefore, able to inactivate bacteria and de-agglomerate bacterial clusters through several physical, mechanical, and chemical effects caused by acoustic cavitation [4,8,10,11].

Recent research on the mechanism of action of ultrasound technology as a means of disinfection by acoustic cavitation has shown the possible role of the glutamate decarboxylase (GAD) system in ultrasound treatment and oxidative stress as well as that of *dnaK* gene of *E. coli* in the general stress response [12]. GAD genes are under the control of many regulators that bind operator sequences [13]. The Δ*gadW* mutant was found to play a role in bacterial resistance to ultrasound treatment when compared with the wild type and other mutants, possibly playing a possible role in ultrasound treatment [12]. Similar studies on the mode of action have been carried out on other disinfection technologies such as plasma, ozone (both producing reactive radicals) and nanomaterials [14,15,16,17]. The most common methods that have been applied include biochemical tests that assess the destruction of food toxins [15] and physiochemical techniques using optical emission spectroscopy that look at bacterial inactivation kinetics in conjugation with radicals produced in situ [16]. Nevertheless, it is imperative to further unravel the mechanism of action of such technologies and specifically of ultrasound to ensure the production of safe and stable ultrasound processed water.

Ultrasound is known to have an impact on the acidity of the treated medium, which may affect bacterial resistance. Previous reports have shown that pathogenic *Escherichia coli* may survive in acidic environments for extended periods [18] and, in fact, an acid-adaptive response in *E. coli* O157:H7 has been previously reported [19,20,21,22]. Inducible resistance mechanisms could increase the resistance of bacteria to acidic conditions [22]. As such, ultrasound at high amplitudes of 37.5 µm has already been shown to enhance survival under acidic conditions, However, further studies are still required to fully understand the underlying mechanism [18].

As of recent, transcriptomics has been used to study the effect of different microbial stresses. Studies of gene expression by looking at RNA transcripts present in cells have increased our knowledge of cell resistance mechanisms and/or regulatory networks that coordinate bacterial stress responses [23]. Different works have described, among others, the modes of action of many antibiotics, mechanisms of bacterial adaptation, and inactivation by heat or by high hydrostatic pressure [23,24]. Furthermore, transcriptional profiling has also shown the induction of general stress responses and proteins after specific methods due to specific cross-resistance phenomena [23,25,26]. This was particularly useful in a study [27] where RNA-Seq was used to identify responses by the *gadEWX* in relation to acid stress. This study highlighted the defence mechanism used by *E. coli* and gave further insight into acid resistance. The study concluded that GadX represses RpoS-mediated transcription of *speG* (spermidine acetyltransferase), which plays a vital role in the control of polyamine concentrations by degradation. This reaction generates protons during acetylation, which results in the repression of such enzymes for the control of proton flow. The regulation of such protons was found to play a role in acid resistance [27]. Further analysis with high-throughput RNA-sequencing (RNA-Seq) methods that are able to capture the global transcriptional response during particular conditions of any organism could allow for the simultaneous analysis of all the regions within the genome, unlike other methods such as RT-PCR, which are still limited to analysing specific and known genomic regions.

The main aim of the current study was to assess the differences between the gene expression of *E. coli* K-12 wild type (WT) and its isogenic mutant ∆*gadW* following exposure to single or sequential cycles of high-power ultrasound treatment. Since ultrasound affects cell viability, the analysis was carried out on a surviving subpopulation of cells after two sequential cycles of ultrasound treatment. The ∆*gadW* mutant was treated similarly to ascertain the role of the GAD system in the stress mitigation response against ultrasound and provides an additional point of contrast for further analysis. Then, RNA-Seq was used to perform a transcriptomic analysis and unravel the mechanism of action of ultrasound treatments. The overall objective was to assess if (sequential) ultrasound treatments result in any transcriptomic changes which can affect the expression of specific genes within *E. coli*.

## 2. Materials and Methods

### 2.1. Bacterial Strains and Preparation of Inoculum

In this study, the bacterial strains used were *E. coli* K-12 wild type MG1655 strain (WT), which is one of the most commonly used laboratory strains with minimal genetic manipulation [28,29], and its isogenic mutant Δ*gadW* obtained from the National Bio-Resource Project, Japan [30,31]. The pure cultures of strains were stored in vials at −80 °C in a freezer using 1% (*v*/*v*) Dimethyl Sulfoxide Solution in Tryptone Soy Broth without dextrose. Before any experiment, pure cultures with isolated colonies were prepared. Under aseptic conditions, a loop from the frozen vial of *E. coli* was streaked onto Tryptone Soya Agar plates (TSA; Oxoid, UK). Following overnight incubation at 37 °C, these pure culture plates were stored at 5 °C and kept for a maximum of 3–4 weeks before further use.

### 2.2. Preparation of the Working Culture

The first subculture was prepared by transferring one isolated colony from the TSA plates to a 10 mL tube containing Tryptic Soy Broth without dextrose (TSB-D) with a sterile loop, followed by subsequent incubation at 37 °C for 24 h. Then, 10 µL from the first subculture was transferred to a small bottle containing 100 mL of TSB-D and incubated at 37 °C for 24 h to reach the stationary phase of growth. After incubation, 10 mL of the culture was taken and centrifuged at 7000× *g* using rotor Sigma (St. Louis, MI, USA) 12151 (centrifuge Sigma—2–6) for 20 min. The supernatant was then discarded, and the pellet was rinsed with 9 mL of Ringer’s solution and centrifuged once more for 20 min. The washing step was repeated twice. The cells were suspended, and 2 mL was transferred into 298 mL of model enriched water. The model enriched water was used to simulate a high nutrient load in water, i.e., to mimic wastewater. This was the sample for the ultrasound treatment. The inoculum was also grown without ultrasound treatment and this served as control.

### 2.3. Ultrasound Treatments

A Hielscher UP200St Ultrasonifier operating at a constant frequency of 26 kHz was used. It was equipped with a generator UP200St g 200 W and a transducer UP200St-T which could be integrated in a sound protection box. A 14 mm sonotrode was used which was suitable to transmit the ultrasound smoothly across a relatively large surface [12]. Ultrasound treatments were applied for 5 min on a continuous mode at 100% intensity Conditions were chosen based on previous studies which resulted in microbial reduction of 0.8 log CFU/mL for the WT and 1.27 log CFU/mL for the Δ*gadW* strain [12].

The model enriched water was produced as described by Antoniadis (2007) and Ayyildiz (2011), that is: peptone 64.0 g; meat extract 44.0 g; urea 12.0 g; K_2_HPO_4_ 11.2 g; NaCl 2.8 g; CaCl_2_.2H_2_O 1.6 g; MgSO_4_.7H_2_O 8 g (St. Louis, MI, USA) made up to 1 L in deionised water. The model enriched water was then autoclaved at 121 °C for 15 min, and its pH was determined to ensure that it was between 7.2–7.8. For each sample, the working solution, consisting of the inoculated enriched water, was transferred into a 500 mL big, jacketed beaker attached to a water pump. The big beaker was carefully disinfected with alcohol between each experiment. Hereafter, the tip of the sonotrode was placed in the centre of the beaker containing 300 mL of bacterial suspension (refer above) with a submerged depth of 2 cm.

In this study, sequential treatment was applied to isolate an ultrasound-resistant bacterial population. After the first treatment, colonies were isolated and re-cultured before a second treatment was ran to assess the impact of ultrasound on microbial-induced resistance. The conditions of the second treatment were the same as the first treatment. Experiment groups that were non-treated (i.e., pre-treatment of ultrasound) or treated with ultrasound were abbreviated as PT or US, respectively. US2 refers to the sequential ultrasound treatment on the same biological replicate.

After each treatment, 1 mL of the sample was transferred to 9 mL Ringer’s solution to perform decimal dilutions which were then plated on TSA plates for plate count enumeration. Bacterial stocks were then stored in a −80 °C freezer for future genetic analysis.

### 2.4. RNA Extraction

RNA extraction was carried out by following the E.Z.N.A.^®^ Bacterial RNA Kit Centrifugation Protocol (Omega BIO-TEK V.5.0, 2018). In summary, the bacteria were cultured from cryovials by inoculating a 10 mL TSB-D broth with a 10 µL sterile loop. The culture was then incubated for 4 h at 37 °C. After incubation, the culture was aspirated and dispensed well with a 1 mL micropipette tip, and 3 mL of culture was transferred into a sterile centrifuge tube. The culture was then centrifuged at 4000× *g* for 10 min at 4 °C. The media was aspirated and discarded. For each pellet, 100 µL of lysozyme solution was added, and the tube was vortexed at maximum speed for 30 s. The manufacturer’s instructions were followed. Two biological samples were analysed for each strain and treatment together with two biological samples for the wild type.

### 2.5. RNA-Seq

RNA-Seq was carried out at Omega Bioservices (Norcross, GA, USA), and thus the standard methods of RNA-Seq on Illumina machines were applied. The method, briefly, is as follows: A QC check for RNA was carried out by using a bioanalyser (Thermo Scientific Nanodrop™ (Waltham, MA, USA, 2018) to achieve the total concentration. The recommended concentration for RNA is ≥ 1 µg for RNA-Seq; however, Illumina kits can successfully amplify RNA from lower amounts starting from 100 ng, provided that more amplification cycles are performed during PCR.

As soon as QC was completed, and the samples were considered free from DNA contamination or RNA degradation, the RNA library was prepared. We used 1 µg of total RNA to prepare Ribo-Zero RNA-Seq libraries. Briefly, rRNA was removed using biotinylated, target-specific oligos combined with Ribo-Zero rRNA removal beads following the Illumina Reference Guide (San Diego, CA, USA). After purification, the RNA was fragmented into small pieces using divalent cations under elevated temperatures. First-strand cDNA syntheses were performed at 25 °C for 10 min, 42 °C for 15 min, and 70 °C for 15 min using random hexamers and ProtoScript II Reverse Transcriptase (New England Biolabs Inc., Ipswich, MA, USA). In a second-strand cDNA synthesis, the RNA templates were removed, and a second replacement strand was generated by incorporation of dUTP to generate double-stranded cDNA. The blunt-ended cDNA was cleaned up from the second-strand reaction mix with beads. The 3′ ends of the cDNA were then adenylated and followed by the ligation of indexing adaptors. PCR (15 cycles of 98 °C for 10 s, 60 °C for 30 s, and 72 °C for 30 s) was used to selectively enrich those DNA fragments that had adapter molecules on both ends to amplify the amount of DNA in the library.

The libraries were quantified and qualified using the D1000 Screen Tape on an Agilent 2200 Tape Station instrument. The libraries were normalized, pooled, and subjected to cluster before pair-read sequencing was performed for 150 cycles on a HiSeq4000 instrument (illumine, Inc., San Diego, CA, USA), according to the manufacturer’s instructions.

### 2.6. Bioinformatics

The analysis was carried out by using Geneious prime 2019. The raw data files from RNA-SEQ analysis were imported into the bioinformatics software. Each data file was then assembled with the latest reference genome, downloaded from the NCBI website: https://www.ncbi.nlm.nih.gov/genome/?term=e%20coli%20k%2012 (accessed on 9 September 2019). Once the contig was created, this was repeated for all the samples. All contigs were then annotated from the reference, and expression levels for all different RNA genes were calculated and compared in order to export the volcano and PCA plots. The RNA genes present were then exported to MS Excel, where the genes were sorted depending on their expression levels. Venn diagrams were also plotted to compare transcribed genes between different treatments, while a heat map (generated by https://software.broadinstitute.org/morpheus, accessed on 20 November 2019) of all the expressed genes was produced to show patterns between the treatments. Finally, the genes obtained were put in Gene Ontology databases (GO) to obtain the mechanisms and KEGG pathways. GO is an international standardised gene functional classification system which used three ontologies: molecular function, cellular component, and biological process. KEGG is one of the accessible pathway analysis databases which allows researchers to understand further biological functions related to gene expression [32].

## 3. Results

Using the Illumina sequencing platform, the raw reads of Table 1 were collected. No poor-quality reads were obtained as a paired score of >30 was achieved for all runs. The reads were subjected to the reference transcriptome to map to the existing gene annotations and were found to have a total of 4497 known genes.

### 3.1. Differentially Expressed Genes (DEGs) and Function Enrichment Analysis

In this study, the criteria of the false discovery rate (FDR) were set as 0.01 and |log2FC| > 1 was used to screen DEGs. The following DEGs were found after comparative analysis between different treatments of different genes. For the Wild Type PT vs. US strain, the majority of genes (35.4%) obtained were up-regulated after treatment. This went down to just 1.8% for the Wild Type PT vs. US2, while for the Wild Type US vs. US2 the majority of genes (41.9%) were down-regulated. On the other hand, for the Δ*gadW* strain, US versus US2 showed a majority of down-regulated genes: 37.2%.

**Figure 1:**
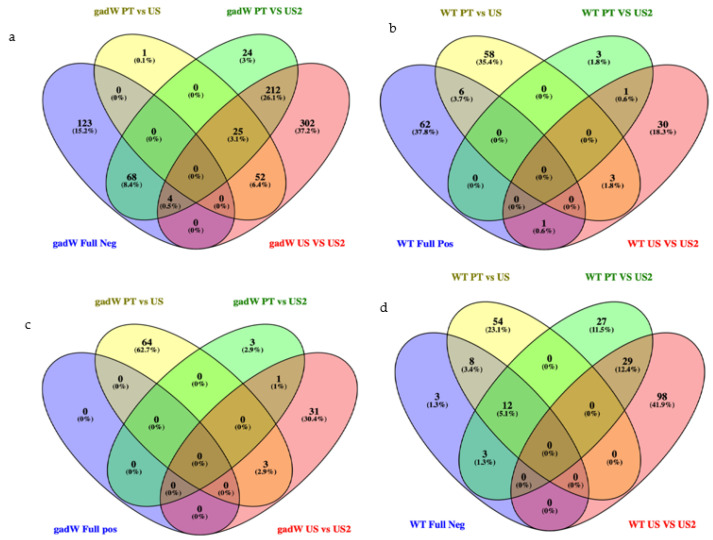
Venn diagrams for the negatively expressed DEGs of *E. coli* wild type over all treatments (**a**), *E. coli* Δ*gadW* (**b**) and of positively expressed DEGs of *E. coli* K-12 wild type (**c**), *E. coli* Δ*gadW* (**d**). The diagrams show the common DEGs between each treatment, together with the overall percentage. Experiment groups that were non-treated (i.e., pre-treatment of ultrasound) or treated with ultrasound were abbreviated as PT or US, respectively. US2 refers to the sequential ultrasound treatment on the same biological replicate.

Venn diagrams revealed unique and common DEG patters between different treatments, as seen in Figure 1.

Venn diagrams are visual representations comparing the different genes obtained under each treatment. The expression of DEGs was found to be significantly different when comparing between the first ultrasound and the second ultrasound treatment, with several genes being affected both positively and negatively by the respective treatment (see Table 2). The different strains, i.e., the WT and Δ*gadW* mutant, also showed different DEGs, indicating that the response of the cells to ultrasound treatment was different, with some genes being directly responsible for defence mechanisms, while others were maybe playing an indirect effect due to cell damage. The table shows that most of the listed genes became negatively expressed as soon as the cell was treated with ultrasound. These genes, which were initially positively expressed, indicate that ultrasound has a negative impact on their expression.

**Figure 2:**
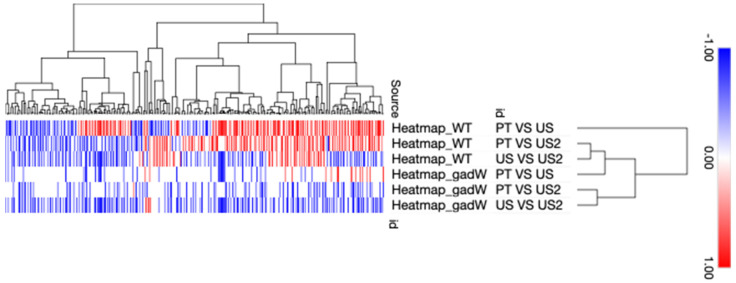
Heat map showing the expression and phylogenetic tree of all the genes in relation to pre-treatment (PT), first ultrasound treatment (US), and sequential ultrasound treatment (US2). Legend is found on top of the diagram, where blue denotes down-regulation and red is up-regulation.

A heat map of all the expressed genes is plotted in Figure 2, where hierarchical clustering summarises the differences between the transcription of each gene and each sample. It shows a gradual decrease in the expression of genes as the cells are treated with ultrasound (as we move from PT to US1 and from PT to US2), which is less pronounced in the *gadW* than the WT. On the other hand, the clustering together of several groups of genes, which are being expressed similarly, indicate which patterns the US treatment has the highest impact on the expression of when compared to the sequential US application.

### 3.2. Gene Ontology Analysis of DEGs

To highlight the categories of the DEGs that were overexpressed for all treatments, all DEGs were annotated to terms in the Gene Ontology (GO) database and were assigned to at least one of the three primary GO categories: biological process—genes which are required for certain biological processes such as respiration and which includes the GAD system; molecular function—genes which are important for molecular function; cellular component—genes that are required for cellular growth or structures. In this case, all the DEGs obtained were used irrespective of the criteria of the false discovery rate (FDR) which was set as 0.01 and |log2FC| > 1. A Fisher’s exact test was then carried out to standardise the results. In the PT vs. US for the WT, 396 DEGs were annotated in the biological process; however, in the case of Δ*gadW*, 385 DEGs for the biological processes, 75 DEGs for the molecular function, and 481 DEGs for the cellular component were annotated. All the DEGs expressed for all treatments are summarised in Table 3.

Further analysis under each category indicates that specific biological processes were positively expressed throughout the US and US2 treatments, whilst others were negatively expressed. When looking at the WT strain, the biological processes for carbohydrate (GO: 0008643) and polyol transport (GO: 0015791) were positively expressed, whilst other biosynthetic and metabolic processes were negatively expressed (such as siderophore biosynthetic processes (GO: 0019290), secondary metabolite biosynthetic processes (GO: 0044550) and others. On the other hand, when looking at Δ*gadW* most of the DEGs were negatively expressed. However, the few biological processes that were consistent were responses to various stress factors such as hyperosmotic response (GO: 0006972), osmotic stress (GO: 0006970), temperature stimulus (Go: 0009266), abiotic stimulus (GO: 0009628), stress (GO: 006950), and stimulus (GO: 0050896).

On the other hand, the WT strain only showed up-regulation in both treatments, in the biological process, and to a lower extend molecular function, and cellular component with all of the DEGs expressed, being related to the respiration cycle (Citrate cycle), construction of flagellum bodies and transporter activity related to the nutrient transfer, respectively. This was not evident in the *gadW* mutant.

Kegg analysis was also carried out to analyse of particular interest was the positive expression of cellular component and various genes related to respiration or flagellum proteins that occurred in the WT strain. From the 67 DEGs in the cellular component group, 55 were related to the respiration cycle while the other 12 are associated with the production of the flagellum.

## 4. Discussion

In this study, the mechanism of action of ultrasound on *E. coli* cells involving various genes were transcribed, showing that expression occurred in a total of 1825 genes. These genes are responsible for mechanisms involved in biological, metabolic, and biosynthetic processes. The isogenic mutant Δ*gadW* was used as this gene was found to play a possible role in ultrasound treatment [12]. The GAD system is known to play an important role in the acid tolerance of bacteria [33,34,35]. However, it has been shown to have an impact on oxidative stress only in *Saccharomyces cerevisiae* [36] and *Francisella tularensis* [37] but not in other organisms. In fact, the GAD system was reported as playing a role in oxidative stress in *E. coli* for the first time in a recent study [12,38].

The transcriptomic analysis provided evidence that *E. coli* K-12 and its isogenic mutant Δ*gadW* responded to ultrasound treatment by altering their gene expression to maintain cellular homeostasis and hence facilitate their survival. As previously reported by Spiteri et al. (2017), the GAD system coupled with the GABA shunt feed into the TCA cycle by affecting succinate and oxoglutarate levels, both of which play a role in oxidative-stress mitigation and thus can confer resistance to oxidant species [39]. This was evident in the DEGs expressed in the WT, as from the 67 genes in the cellular component, 55 were related to the respiration cycle, which is related to the GAD system, whilst the other 12 were related to the production of the flagellum. These genes were all positively expressed and involved in the production of the flagellum, which could easily be as a form of repair mechanism, or otherwise involved in better cell movement [38]. One must keep in mind that the production of flagella may be quite an energy-intensive process and may not be promoted under stress conditions. This shows that *gadW* is involved in the regulation of various cellular functions apart from that of the GAD system. Seo et al. (2015) observed that repression of the *speG* gene protects the cell from acidic damage, which is in line with the current study in which the *speG* gene of the WT in both PT/US2 and US/US2 was also down-regulated. This further indicates the role of the *gadEWX* regulation network in acid resistance. On the other hand, no expression in the isogenic mutant of the *speG* gene was found in any of the treatments, corroborating the findings mentioned in the study above.

These variants can be further assessed by studies of gene expression and regulation mechanisms. The observed mutation effects of ultrasound treatment on the *E. coli* K-12 bacteria occurred at four different areas of the genome: the IS5 transposase area, the araBAD transcription site, the side tail fibre, and the intragenic valX site. Although they may not have a significant effect on the bacteria, they indicate how environmental stresses influence the bacteria. In the first step of the treatment a single mutation just outside the transcription site of the araBAD site was observed in both biological replicates of the wild type bacteria. This site was reported to regulate the sigma 70 factor, which is essential for the normal growth of the cell, especially during exponential growth. This was also found to correlate well with a phenotypic observation wherein the expression of motility of the bacteria cells was delayed in the wild type bacteria. The results also show that this change was again reverted with the second treatment, where some replicates showed delayed motility. The observed phenomenon could also be related to the fact that a mutation in the part coding for the side tail fibre was reported, which was found to alter microbial colonisation. This may indicate that single US treatment should be sufficient for reducing the viability of bacterial cells. Further sequential treatment should only be advocated if bacterial cells need to be totally killed off in order to avoid cell response and repair. On the other hand, an insertion mutation in the IS5 was observed outside the gene IS21, just upstream the oppA gene. This is an indication that no major effect on the genes occurred. However, one must point out that the oppA gene is an essential gene for survival under heat stress and as such could be a defence mechanism, which merits further investigation [40].

As mentioned in several studies, succinate transport is carried out by dicarboxylic acid transport [41,42,43,44,45]. It is regulated by the gene *dctA*, which was upregulated in the WT when treated with ultrasound. However, it has to be highlighted that this was only significantly different in the single treatment, rather than the double-treated cells. This was also evident when comparing the expression of the US treatment with the US2 treatment, which showed a down-regulation of this gene.

Furthermore, since specific oxidase genes, *sodA* and *sodC*, were positively transcribed, this may indicate that the bacterial cells were under oxidative stress [46]. Both genes were positively expressed when the treated cells were compared with the non-treated cells; however, when we compared the cells treated for the second time with the non-treated cells, only *sodC* was overexpressed.

The sigma factor RpoS can be activated and regulates the expression of genes in response to general stress [46,47,48]. An interesting note to point out is that under stress, RpoS induces the mutagenic repair of DNA breaks through a network of genes [49], some of which were positively expressed in this study: *yifE*, *nuoG*, *hemL*, *hdfR*, *cspC*, *hfq*, and *sdhB*. This suggests that ultrasound treatment induces similar effects to those elicited by superoxide groups, whilst also giving rise to DNA damage. The expression of the *hfq* gene is particularly interesting as it was found to play an essential role in the stress response since it regulates two major transcription factors: σS and σE [50,51,52]. These genes sense stress and promote the repair of DNA of double-stranded breaks (DSBs). σS controls general- or starvation-stress responses while σE promotes membrane-protein stress responses [49]. Regulator genes such as *rpoS* exhibit the capability to control several pathways under stress by regulating many proteins involved in the central metabolic system, indicating that the cell would be trying to promote resources for the continued survival of the cell. This was consistent with other studies, where transcriptomic changes were studied under different stresses [23,25,46,52].

In *E. coli* cells, the activity of *CpxA* is influenced by the composition of membrane lipids [47]. In our results, it is evident that both CPX and LPX systems do not seem to be playing a significant (0.01 significance) role in the WT strain, as the systems come in play in the *gadW* mutant. In all cases for the isogenic mutant, both the *cpxP* and *lpxC* systems were down-regulated. On the other hand, the study of Li et al. (2018) showed that these systems were up-regulated in cases where cells were treated with prolonged cold stress. The down-regulation of the ultrasound-treated cells shows the *CpxP* provides a negative feedback regulator for the pathway and thus is an effector of the stress response. This results in displacement of *CpxP* from *CpxA* and thus activation of the Cpx response [53].

The cpx system is complex, and the *cpxA* serves as a stress response alongside facilitating the assembly of pili. Furthermore, also being a periplasmic chaperone, it could be induced directly in response to damage to pili [54] caused by ultrasound treatment. On the other hand, another gene, *lpxA*, which is required for cell division, growth under hypoosmotic conditions, and viability, in general, seem to have a role in conjunction with the cpx system in cell integrity on the loss of outer membrane impermeability [55].

The Cpx response, together with a *gadE* transcriptional activator, gives rise to a *lpx* response. The *lpxD* gene is generally activated by the presence of antibiotics in the formation of lipids and is an essential gene [28,55]. However, for this to be transcribed, the *lpxD* needs an activated Cpx response together with the transcriptional activator, which was absent in the *gadW* mutant, thus showing a negative transcription. However, this process was not found to be crucial in the protection of the WT strain from ultrasound as no significant expression was observed in this strain, unlike that found by Li. et al. (2018) in the protection of cold shock.

## 5. Conclusions

In conclusion, several DEGs were identified in this study, with many identified as playing a role in stress in *E. coli* K-12 by using throughput transcriptome sequencing technology. *E. coli* K-12 also appears to have gene expression involved in protection from pro-longed harm, in respiration for increased growth, production of flagellum and pili, and also expression in various protection mechanisms such as sigma S and E. Furthermore, membrane responses also indicate the widespread effect of ultrasound treatment on the cell. Although this study indicates that ultrasound has an impact on this bacteria, which in turn is changes its mechanisms for enhanced resistance, further work would provide a better understanding of what resistance is being permanently shown in future generations of the bacteria, providing additional light on the use of disinfection in industry. Further studies could focus on assessing other mutants (e.g., in the Keio collection—the Keio collection is comprised of 3985 deletions in duplicate (7970 total) of *E*. *coli* K-12 strain BW25113 [56], a strain with a well-defined pedigree that has not been subjected to mutagens) in order to test various other global stress regulators and identify if other stress responses are involved. The above stress assessments could also be tested in biofilms to identify differences in responses.

## Figures and Tables

**Table 1:** Summary of the raw reads for each sequencing run per sample. (WT—Wild type; PT—No US treatment; US—After US treatment; US2—Sequential US treatment on same biological replicate).

Strain with Treatment	Replicate 1		Replicate 2	
	Unique Reads	Duplicate Reads	Unique Reads	Duplicate Reads
WT PT	1,907,618	9,782,951	1,313,674	10,376,895
WT US	1,638,411	8,729,411	2,259,703	8,107,832
WT US2	2,510,105	7,998,899	2,896,851	7,612,563
Δ*gadW* PT	1,903,140	8,570,279	2,348,814	8,124,605
Δ*gadW* US	2,247,793	8,586,961	2,727,471	8,107,283
Δ*gadW* US2	2,466,568	7,854,287	2,819,178	7,501,677

**Table 2:** Comparative table for specific DEGs of WT over three treatments showing the fold change, Log2FC, and the *p*-value. *p*-values in bold are significantly different. Statistical analysis used 95% confidence limits (WT—Wild type; PT—No US treatment; US—After US treatment; US2—Sequential US treatment on same biological replicate).

	Fold Change log2FC			*p*-Value			
DEGs	PT vs. US	PT vs. US2	US vs. US2	PT vs. US	PT vs. US2	US vs. US2	Annotation
*rpoS*	0.510	0.466	−0.076	**<0.0001**	**<0.0001**	0.19477937	General stress
*sodC*	1.182	1.013	−0.165	**<0.0001**	**<0.0001**	0.3052228	Oxidase gene
*sodA*	1.164	−0.170	−1.365	**<0.0001**	**<0.0001**	**<0.0001**	Oxidase gene
*SodB*	0.438	0.270	−0.195	**<0.0001**	0.0297	0.0469	Oxidase gene
*yifE*	0.381	−0.959	−1.367	**0.00155**	**<0.0001**	**<0.0001**	Stress-induced mutagenesis
*nuoG*	0.521	0.449	−0.099	**<0.0001**	**<0.0001**	0.326	Stress-induced mutagenesis
*hemL*	0.654	−0.202	−0.883	**<0.0001**	0.0941	**<0.0001**	Stress-induced mutagenesis
*hdfR*	0.713	0.496	−0.250	**<0.0001**	**<0.0001**	0.0479	negatively expresses flagellar master
*cspC*	0.811	−0.246	−1.101	**<0.0001**	0.0495	**<0.0001**	Stress-induced mutagenesis
*hfq*	0.855	−0.151	−1.039	**<0.0001**	**0.00530**	**<0.0001**	Stress-induced mutagenesis
*sdhB*	1.552	0.334	−1.214	**<0.0001**	0.0503	**<0.0001**	Stress-induced mutagenesis
*lpxD*	0.265	0.247	−0.060	0.0468	0.0795	0.601	lipid biosynthesis
*dctA*	1.254	0.522	−0.761	**<0.0001**	**<0.0001**	**<0.0001**	required for dicarboxylate transport
*speG*		−1.003	−1.061		**<0.0001**	**<0.0001**	Protection against polyamine toxicity
*cpxP*	0.397	−0.164	−0.595	**<0.0001**	**<0.0001**	**<0.0001**	Auxiliary protein in the CpX two-component envelope stress response system
*lpxC*	0.399		−0.436	**<0.0001**		**<0.0001**	Develops new antibacterial agents

**Table 3:** Comparative table showing the expressed DEGs of both mutants with their respective pathways. All DEGs listed were significant at *p* < 0.05 (WT—Wild type; PT—No US treatment; US—After US treatment; US2—Sequential US treatment on same biological replicate).

		Biological Process		Molecular Function		Cellular Component	
Strain	Treatment	Positive Expression	Negative Expression	Positive Expression	Negative Expression	Positive Expression	Negative Expression
WT	PT vs. US	396	N/A	N/A	N/A	N/A	N/A
	PT vs. US2	301	N/A	11	N/A	52	85
	US vs. US2	41	220	14	N/A	15	90
*gadW*	PT vs. US	104	281	N/A	75	N/A	481
	PT vs. US2	N/A	2585	N/A	1186	N/A	2582
	US vs. US2	N/A	4714	N/A	1634	N/A	4122

## Data Availability

Data are contained within the article and Supplementary Materials.

## Supplementary Materials

The following supporting information can be downloaded at: https://www.mdpi.com/article/10.3390/microorganisms11112768/s1, Table S1: qPCR reaction composition, Table S2: qPCR cycle conditions, Table S3: Primer sequences used for the measurement of specific genes in *E. coli* K-12, were designed using the NCBI ‘pick primers’ feature and validated using primer blast, Figure S1: Comparison of gene expression after ultrasound treatment. (A) Pre-Treatment (PT) vs first ultrasound treatment (US), (B) Pre-Treatment (PT) vs second ultrasound treatment (US2) and (C) first ultrasound treatment (US) vs second ultrasound treatment (US2) gene expression was the *E. coli* K-12 wildtype MG1655 strain transcriptome measured with RNA sequencing and compared to that of rpoS, sodC, sodA, nuoG and sdhB measured with qPCR. Data are presented as mean (±SD) of two biological replicates. Data were analysed with Two-way ANOVA with Sidak’s post-hoc modification. Data significantly different for each gene between the two techniques is denoted with an ‘*’, where ‘*’ *p* < 0.05 and ‘***’ *p* < 0.001.

## References

[1] Cesaro A., Belgiorno V. (2016). Removal of Endocrine Disruptors from Urban Wastewater by Advanced Oxidation Processes (AOPs): A Review. Open Biotechnol. J..

[2] Han C., Andersen J., Pillai S., Fagan R., Falaras P., Byrne J., Dunlop P., Choi H., Jiang W., O’Shea K. (2013). Chapter green nanotechnology: Development of nanomaterials for environmental and energy applications. ACS Symp. Ser..

[3] Gibson J.H., Yong D.H.N., Farnood R.R., Seto P. (2008). A Literature Review of Ultrasound Technology and Its Application in Wastewater Disinfection. Water Qual. Res. J..

[4] Sango D.M., Abela D., McElhatton A., Valdramidis V. (2014). Assisted ultrasound applications for the production of safe foods. J. Appl. Microbiol..

[5] Madge B., Jensen J. (2002). Disinfection of wastewater using a 20-kHz ultrasound unit. Water Env. Res..

[6] Oyib D.H. (2010). Ultrasound in Water Treatment: Suppressing Algal Growth and Biofilm Formation. http://www.iwaponline.com/w21/01103/w21011030052.htm.

[7] Naddeo V., Landi M., Belgiorno V., Napoli R. (2009). Wastewater disinfection by combination of ultrasound and ultraviolet irradiation. J. Hazard. Mater..

[8] Vasilyak L.M. (2010). Ultrasound application in systems for the disinfection of water. Surf. Eng. Appl. Electrochem..

[9] Drakopoulou S., Terzakis S., Fountoulakis M., Mantzavinos D., Manios T. (2009). Ultrasound-induced inactivation of gram-negative and gram-positive bacteria in secondary treated municipal wastewater. Ultrason. Sonochem..

[10] Antoniadis A., Poulios I., Nikolakaki E., Mantzavinos D. (2007). Sonochemical disinfection of municipal wastewater. J. Hazard. Mater..

[11] Broekman S., Pohlmann O., Beardwood E., de Meulenaer E.C. (2010). Ultrasonic treatment for microbiological control of water systems. Ultrason. Sonochem..

[12] Spiteri D., Chot-Plassot C., Sclear J., Karatzas K., Scerri C., Valdramidis V. (2017). Ultrasound processing of liquid system(s) and its antimicrobial mechanism of action. Lett. Appl. Microbiol..

[13] Johnson M. (2011). Understanding the Regulation of Acid Resistance in E. coli Using Whole Genome Techniques.

[14] Nath A., Mukhim K., Swer T., Dutta D., Verma N., Deka B., Gangwar B. (2014). A Review on the Application of Nanotechnology in Food Processing and Packaging. J. Food Prod. Dev. Packag..

[15] Mahapatra A.K., Muthukumarappan K., Julson J.L. (2005). Applications of Ozone, Bacteriocins and Irradiation in Food Processing: A Review. Crit. Rev. Food Sci. Nutr..

[16] Perni S., Shama G., Hobman J.L., Lund P.A., Kershaw C.J., Hidalgo-Arroyo G.A., Penn C.W., Deng X.T., Walsh J.L., Kong M.G. (2007). Probing bactericidal mechanisms induced by cold atmospheric plasmas with *Escherichia coli* mutants. Appl. Phys. Lett..

[17] Laroussi M. (1996). Sterilization of contaminated matter with an atmospheric pressure plasma. IEEE Trans. Plasma Sci..

[18] Patil S., Bourke P., Kelly B., Frías J.M., Cullen P. (2009). The effects of acid adaptation on *Escherichia coli* inactivation using power ultrasound. Innov. Food Sci. Emerg. Technol..

[19] Leyer G.J., Wang L.L., A Johnson E. (1995). Acid adaptation of *Escherichia coli* O157:H7 increases survival in acidic foods. Appl. Environ. Microbiol..

[20] Berry E.D., Cutter C.N. (2000). Effects of Acid Adaptation of *Escherichia coli* O157:H7 on Efficacy of Acetic Acid Spray Washes to Decontaminate Beef Carcass Tissue. Appl. Environ. Microbiol..

[21] Šeputienė V., Daugelavičius A., Sužiedėlis K., Sužiedėlienė E. (2006). Acid response of exponentially growing *Escherichia coli* K-12. Microbiol. Res..

[22] Tosun H. (2014). The Effect of Acid Adaptation Conditions on Acid Tolerance Response of *Escherichia coli* O157: H7. Turkish J. Biol..

[23] Chueca B., Pagán R., García-Gonzalo D. (2015). Transcriptomic analysis of *Escherichia coli* MG1655 cells exposed to pulsed electric fields. Innov. Food Sci. Emerg. Technol..

[24] Wecke T., Mascher T. (2011). Antibiotic research in the age of omics: From expression profiles to interspecies communication. J. Antimicrob. Chemother..

[25] Carruthers M.D., Minion C. (2009). Transcriptome analysis of *Escherichia coli* O157:H7 EDL933 during heat shock. FEMS Microbiol. Lett..

[26] Shin J.-H., Kim J., Kim S.-M., Kim S., Lee J.-C., Ahn J.-M., Cho J.-Y. (2010). σB-dependent protein induction in Listeria monocytogenes during vancomycin stress. FEMS Microbiol. Lett..

[27] Seo S.W., Kim D., O’brien E.J., Szubin R., Palsson B.O. (2015). Decoding genome-wide GadEWX-transcriptional regulatory networks reveals multifaceted cellular responses to acid stress in *Escherichia coli*. Nat. Commun..

[28] Dwyer D.J., A Kohanski M., Collins J.J. (2009). Role of reactive oxygen species in antibiotic action and resistance. Curr. Opin. Microbiol..

[29] Blattner F.R., Plunkett G., Bloch C.A., Perna N.T., Burland V., Riley M., Collado-Vides J., Glasner J.D., Rode C.K., Mayhew G.F. (1997). The Complete Genome Sequence of *Escherichia coli* K-12. Science.

[30] Baba T., Ara T., Hasegawa M., Takai Y., Okumura Y., Baba M., Datsenko K.A., Tomita M., Wanner B.L., Mori H. (2006). Construction of *Escherichia coli* K-12 in-frame, single-gene knockout mutants: The Keio collection. Mol. Syst. Biol..

[31] Yamamoto N., Nakahigashi K., Nakamichi T., Yoshino M., Takai Y., Touda Y., Furubayashi A., Kinjyo S., Dose H., Hasegawa M. (2009). Update on the Keio collection of *Escherichia coli* single-gene deletion mutants. Mol. Syst. Biol..

[32] Kanehisa M., Araki M., Goto S., Hattori M., Hirakawa M., Itoh M., Katayama T., Kawashima S., Okuda S., Tokimatsu T. (2007). KEGG for linking genomes to life and the environment. Nucleic Acids Res..

[33] Smith D.K., Kassam T., Singh B., Elliott J.F. (1992). *Escherichia coli* has two homologous glutamate decarboxylase genes that map to distinct loci. J. Bacteriol..

[34] Feehily C., Karatzas K. (2012). Role of glutamate metabolism in bacterial responses towards acid and other stresses. J. Appl. Microbiol..

[35] Paudyal R., Karatzas K. (2016). Stress adaptation of Listeria monocytogenes in acidic ready-to-eat products A2—Kotzekidou, Parthena. Food Hygiene and Toxicology in Ready to Eat Foods.

[36] Klepsch M., Kovermann M., Löw C., Balbach J., Permentier H., Fusetti F., de Gier J., Slotboom D., Berntsson R.P.-A. (2011). *Escherichia coli* Peptide Binding Protein OppA Has a Preference for Positively Charged Peptides. J. Mol. Biol..

[37] Coleman S.T., Fang T.K., Rovinsky S.A., Turano F.J., Moye-Rowley W.S. (2001). Expression of a Glutamate Decarboxylase Homologue Is Required for Normal Oxidative Stress Tolerance in Saccharomyces cerevisiae. J. Biol. Chem..

[38] Boura M., Brensone D., Karatzas K.A. (2019). A novel role for the glutamate decarboxylase system in Listeria monocytogenes; protection against oxidative stress. Food Microbiol..

[39] Janausch I., Zientz E., Tran Q., Kröger A., Unden G. (2001). C4-dicarboxylate carriers and sensors in bacteria. Biochim. Biophys. Acta (BBA) Bioenerg..

[40] Ramond E., Gesbert G., Rigard M., Dairou J., Dupuis M., Dubail I., Meibom K., Henry T., Barel M., Charbit A. (2014). Glutamate Utilization Couples Oxidative Stress Defense and the Tricarboxylic Acid Cycle in Francisella Phagosomal Escape. PLoS Pathog..

[41] Groeneveld M., Weme R.G.J.D.O., Duurkens R.H., Slotboom D.J. (2010). Biochemical Characterization of the C _4_ -Dicarboxylate Transporter DctA from *Bacillus subtilis*. J. Bacteriol..

[42] Sá-Pessoa J., Paiva S., Ribas D., Silva I.J., Viegas S.C., Arraiano C.M., Casal M. (2013). SATP (YaaH), a succinate–acetate transporter protein in *Escherichia coli*. Biochem. J..

[43] Karinou E., Hoskisson P.A., Strecker A., Unden G., Javelle A. (2017). The *E. coli* dicarboxylic acid transporters DauA act as a signal transducer by interacting with the DctA uptake system. Sci. Rep..

[44] Lo B., Khalil T., Sanwal M. (1972). Transport of Succinate in *Escherichia*. Biol. Chem..

[45] Gottesman S. (2019). Trouble is coming: Signaling pathways that regulate general stress responses in bacteria. J. Biol. Chem..

[46] Li Y., Zhou D., Hu S., Xiao X., Yu Y., Li X. (2018). Transcriptomic analysis by RNA-seq of *Escherichia coli* O157:H7 response to prolonged cold stress. LWT.

[47] Al Mamun A.A.M., Lombardo M.-J., Shee C., Lisewski A.M., Gonzalez C., Lin D., Nehring R.B., Saint-Ruf C., Gibson J.L., Frisch R.L. (2012). Identity and Function of a Large Gene Network Underlying Mutagenic Repair of DNA Breaks. Science.

[48] Brown L., Elliott T. (1996). Efficient translation of the RpoS sigma factor in Salmonella typhimurium requires host factor I, an RNA-binding protein encoded by the hfq gene. J. Bacteriol..

[49] Muffler A., Fischer D., Hengge-Aronis R. (1996). The RNA-binding protein HF-I, known as a host factor for phage Qbeta RNA replication, is essential for rpoS translation in *Escherichia coli*. J. Bone Jt. Surg..

[50] Guisbert E., Rhodius V.A., Ahuja N., Witkin E., Gross C.A. (2007). Hfq Modulates the σ ^E^ -Mediated Envelope Stress Response and the σ ^32^ -Mediated Cytoplasmic Stress Response in *Escherichia coli*. J. Bacteriol..

[51] King T., Lucchini S., Hinton J.C.D., Gobius K. (2010). Transcriptomic Analysis of *Escherichia coli* O157:H7 and K-12 Cultures Exposed to Inorganic and Organic Acids in Stationary Phase Reveals Acidulant- and Strain-Specific Acid Tolerance Responses. Appl. Environ. Microbiol..

[52] Karp P.D., Weaver D., Paley S., Fulcher C., Kubo A., Kothari A., Krummenacker M., Subhraveti P., Weerasinghe D., Gama-Castro S. (2014). The EcoCyc Database. EcoSal Plus.

[53] Allen M.J., White G.F., Morby A.P. (2006). The response of *Escherichia coli* to exposure to the biocide polyhexamethylene biguanide. Microbiology.

[54] Kelly T., Stachula S., Raetz C., Anderson M. (1993). The firA gene of *Escherichia coli* encodes UDP-3-O-(R-3-hydroxymyristoyl)- glucosamine N-acyltransferase. The third step of endotoxin biosynthesis. J. Biol. Chem..

[55] Oliveros J.C. Venny. An Interactive Tool for Comparing Lists with Venn Diagrams. http://bioinfogp.cnb.csic.es/tools/venny/index.html.

[56] Datsenko K., Wanner B. (2000). One-step inactivation of chromosomal genes in *Escherichia coli* K-12 using PCR products. Proc. Natl. Acad. Sci. USA.

