# Supplementary material for: Escherichia coli K-12 Transcriptomics for Assessing the Mechanism of Action of High-Power Ultrasound
Source: Microorganisms. 2023 Nov 14;11(11):2768. doi: 10.3390/microorganisms11112768 (PMC10673019; doi:10.3390/microorganisms11112768)
Supplement: Supplementary file 1 [file microorganisms-11-02768-s001.zip › microorganisms-2569752-supplementary.pdf]

Appendix 1 – qPCR

The same replicate samples, used for RNA-SEQ, which were stored in the -80 °C were processed for qPCR. Two housekeeping genes, *mdoG* and *dapA* were selected together with the five selected Gene of Interest (GOI). These genes were selected at random from the expressed RNA-SEQ data namely: *rpoS*, *sodC*, *sodA*, *yifE* and *sdhB*. RNA extraction was carried out following protocol described in section 2.4 above. The cDNA, using FIREScript® RT cDNA synthesis KIT (Solis Biodyne, Estonia) was then transcribed, and a calibration curve performed utilizing three 10-fold dilutions. All genes were analyzed in duplicate. SOLIScript® 1-step SolisGreen® kit (Solis Biodyne, Estonia) was used, and the manufacturer's instructions were followed. These are summarised in Table S1 and S2, below. All Primers used were designed from NCBI-Blast website and summarised in Table S3. Once all the Ct values were obtained, data was normalized, and the deltaCT value was calculated. The log RPKM from RNA-SEQ was then compared with the data obtained for RNA-SEQ.

Five random genes were selected, and qPCR was carried out to identify the expression. From the results obtained, it can be identified that RNA-SEQ results obtained, correlated well with the qPCR analysis. Figure. S1 summarizes the results. The gene expression as assessed by qPCR and RNAseq using Graphpad Prism 8 and compared using Two-way ANOVA.The same pattern of expression was observed with both qPCR and RNA-SEQ. Although some statistically significant changes were observed between the two methods, software. the trends followed by gene expression were the same, *i.e.*, genes measured as upregulated were measured consistently with both qPCR and RNASeq, with a similar magnitude Any statistically significant differences can therefore be attributed to the use of different techniques.

TableS1: qPCR reaction composition.

Component	Volume (µL)	Concentration
40x One-step SOLIScript® Mix	0.5	1X
5x One-step SolisGreen® Mix	4.0	1X
Forward Primer (10uM)	0.8	400 nM
Reverse Primer (10uM)	0.8	400 nM
Template DNA	5.0	
Nuclease – free water	9.4	

TableS2: qPCR cycle conditions.

Step	Temperature (°C)	Time	Cycles
Reverse Transcription	50	15 min	1
Enzyme activation	95	10 min	1
Denaturation	95	15 s	40
Annealing	60	20 s	
Extension	72	20 s	

Table S3: Primer sequences used for the measurement of specific genes in E. coli K-12, were designed using the NCBI 'pick primers' feature and validated using primer blast.

Primer	Sequence 5' – 3'
rpoS	F-CGAAAAAGCGTTGCTGGACA
	R-GCTCGAACAGCCATTTGACG
sodC	F-CCACCAAAGATGGCAAAGCC
	R-CACCTTCTGGCCCTTCATGT
sodA	F-ACTGGCGGTGGTTTCTACTG
	R- CCAGTTCACCACGTTCCAGA
yifE	F-ACTGCTTGAGCGTCATGGTT
	R-CTGCTTCTGTCACTGGCTCA
sdhB	F-GTCTGGCCTGTATTACCCCG
	R-GTCTACCACCAAATCGCGGA
mdoG	F-GCAGGTCAGGTTTATGGCCT
	R-CAGTTGGTTTTGGACGCTCG
dapA	F-CGGTGATTGGTGTCATTGGC
	R-TCCAATCCCGGTGAAATGGG

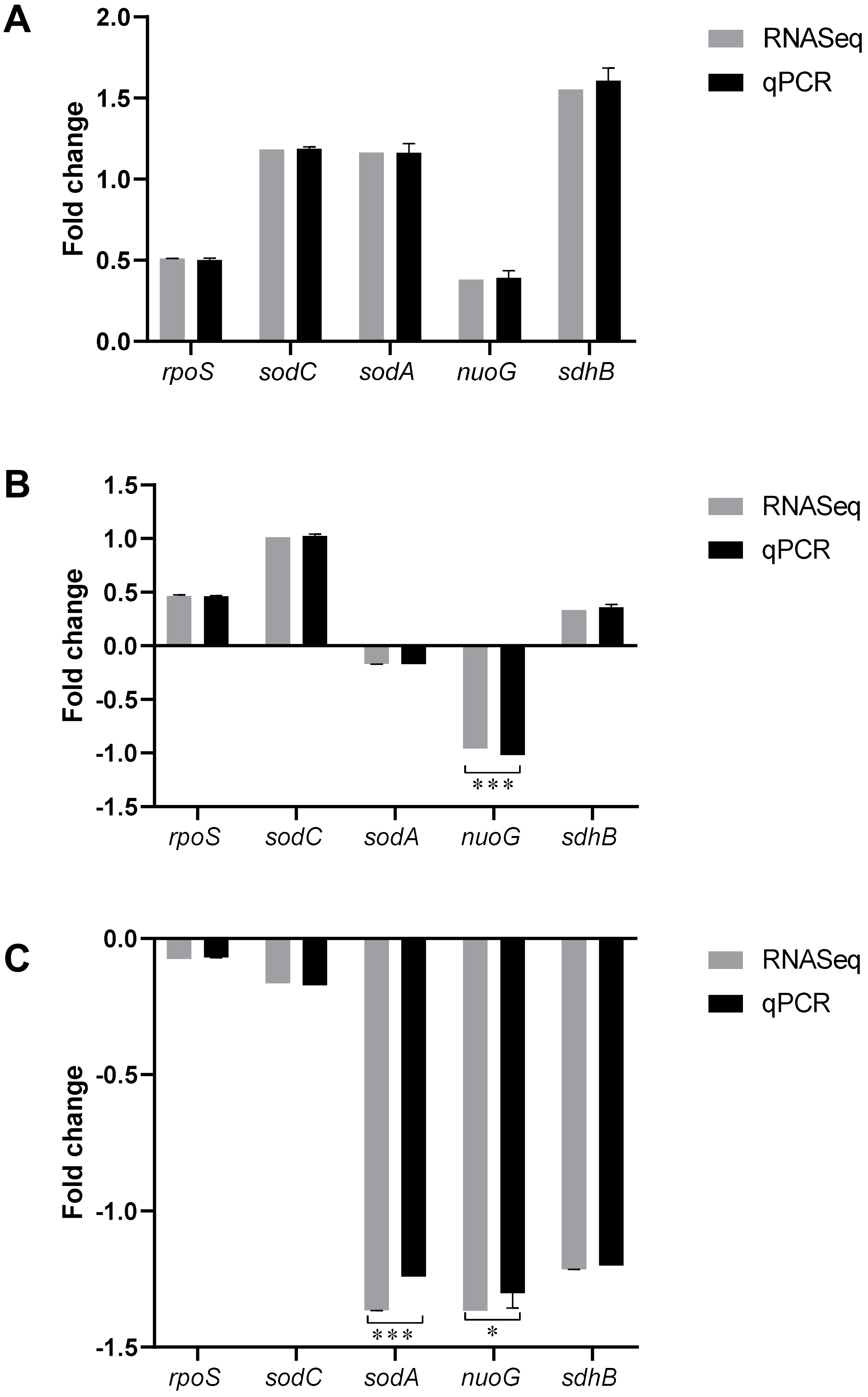
A
B
C
Fold change
2.0
1.5
1.0
0.5
0.0
-0.5
-1.0
-1.5
rpoS
sodC
sodA
nuoG
sdhB
RNASeq
qPCR
***
*

Figure S1. Comparison of gene expression after ultrasound treatment. (A) Pre-Treatment (PT) vs first ultrasound treatment (US), (B) Pre-Treatment (PT) vs second ultrasound treatment (US2) and (C) first ultrasound treatment (US) vs second ultrasound treatment (US2) gene expression was the *E. coli K*-12 wild type MG1655 strain transcriptome measured with RNA sequencing ( ) and compared to that of *rpoS, sodC, sodA, nuoG* and *sdhB* measured with qPCR ( ). Data are presented as mean (±SD) of two biological replicates. Data were analysed with Two-way ANOVA with Sidak's post-hoc modification. Data significantly different for each gene between the two techniques is denoted with an '*', where '*' $p < 0.05$ and '***' $p < 0.001$.
